# Exploring the Breastfeeding Desires and Decision-Making of Women Living with HIV in the Netherlands: Implications for Perinatal HIV Management in Developed Countries

**DOI:** 10.1089/bfm.2023.0004

**Published:** 2023-05-17

**Authors:** Vera E. Bukkems, Renee N.N. Finkenflügel, Karin Grintjes, Manon Marneef, Martine de Haan, Inga Mielitz, Astrid van Hulzen, Casper Rokx, Elisabeth van Leeuwen, Jeannine F. Nellen, David M. Burger, Angela Colbers

**Affiliations:** ^1^ Department of Pharmacy, Research Institute for Medical Innovation, Radboud University Medical Center, Nijmegen, the Netherlands.; ^2^ Hiv Vereniging, Amsterdam, the Netherlands.; ^3^ Department of Internal Medicine, Radboud University Medical Center, Nijmegen, the Netherlands.; ^4^ ShivA, Amsterdam, the Netherlands.; ^5^ Department of Internal Medicine, Isala Hospital, Zwolle, the Netherlands.; ^6^ Department of Internal Medicine, Erasmus MC, Rotterdam, the Netherlands.; ^7^ Department of Medical Microbiology and Infectious Diseases, Erasmus MC, Rotterdam, the Netherlands.; ^8^ Department of Obstetrics, Academic Medical Center, University of Amsterdam, Amsterdam, the Netherlands.; ^9^ Division of Infectious Diseases, Tropical Medicine and AIDS, Department of Internal Medicine, Academic Medical Center, University of Amsterdam, Amsterdam, the Netherlands.

**Keywords:** antiretroviral agents, breastfeeding, HIV, shared decision-making, patient-centered care, mother-to-child transmission

## Abstract

**Introduction:**

Guidelines in high-income countries recommend women living with human immunodeficiency virus (HIV) to formula feed their newborns, because the possibility of mother-to-child-transmission of HIV during breastfeeding cannot be ruled out. It is an ongoing debate if the possible transmission risk outweighs the medical, cultural, psychological, and social importance of breastfeeding in women stable on current first-line suppressive antiretroviral regimens. The study aim was to explore breastfeeding desires and decision-making of immigrant and nonimmigrant women living with HIV in the Netherlands.

**Method:**

A questionnaire was administered orally or online to 82 women living with HIV in the Netherlands. The breastfeeding desires of the participants were collected as categorical data, and breastfeeding decision-making and willingness to adhere to additional monitoring were collected on a 5-point Likert scale. Categorical data were presented as proportions, and Likert scale data were presented in Likert scale bar plots.

**Results:**

Seventy-one percent of the participants expressed a desire to breastfeed in the future. The most important factors influencing decision-making to breastfeed were the chance of transmission of HIV to the infant and the advice by the doctor or nurse practitioner. Of the participants, 42% expressed their interest in breastfeeding with a <1/100 transmission risk. More than half of the participants expressed their interest to breastfeed with additional monitoring.

**Conclusions:**

A substantial proportion of the women living with HIV in the Netherlands has a desire to breastfeed, of which the majority are willing to adhere to additional monitoring to do so.

## Introduction

In contrast to low- and middle-income countries, guidelines in high-income countries recommend women living with human immunodeficiency virus (HIV) to formula feed their newborns because mother-to-child-transmission (MTCT) of HIV during breastfeeding cannot be ruled out completely.^1,2^ Recent guidelines, including European AIDS Clinical Society (EACS) and Department of Health and Human Services (DHHS), also acknowledge that women may want to breastfeed, and some physicians in the Netherlands currently support breastfeeding as an alternative to formula feeding for HIV-infected women with prolonged viral suppression.^1–3^

It is an ongoing debate if the possible MTCT risk outweighs the medical, cultural, psychological, and social importance of breastfeeding in women stable on suppressive antiretroviral regimens, especially with newer and more potent regimens available now.^4–6^ The MTCT rate is low (<1%) when women have an undetectable viral load postpartum, as shown by the investigators of several studies in low-income setting.^7–10^ This MTCT risk is associated with maternal antiretroviral drug adherence.^11,12^ It is unclear if there is a risk of MTCT in adherent women on successful long-term antiretroviral treatment living in high-income countries, and if undetectable = untransmissible (U = U) applies for these women.

The previously mentioned studies were carried out in low-income settings, and transmission occurred only in women on inferior therapy, in nonadherent women or in women starting therapy during late pregnancy.^7–10^ Only a limited number of cases of breastfeeding women living with HIV in high-income setting have been reported. No MTCT occurred in these cases, but the number of cases is too limited to draw conclusions on the possible MTCT risk; however, the consensus is that among treatment-adherent women, the transmission rate is probably <1%, but not necessarily zero.^13–15^

There is also limited information on the other side of the story: the attitude of women living with HIV in high-income countries toward the cultural, psychological, and social importance of breastfeeding. Previously, investigators performing explorative studies in Canada and the United Kingdom showed that abstaining from breastfeeding came with considerable emotional costs.^16,17^ In addition, one-third to half of the study participants from studies in the United Kingdom and Switzerland indicated that they would like to breastfeed despite having HIV.^18,19^ Little is known about the breastfeeding desires among women living with HIV in the Netherlands. Also, the prevalence of breastfeeding despite medical advice to formula feed is unknown. This has only been asked to health care providers in the United States, of whom 29% reported to care for a woman living with HIV who has or had breastfed.^20^

In this study, we aim to explore breastfeeding preferences and decision-making of immigrant and nonimmigrant women living with HIV in the Netherlands. With this information we will be able to offer tailor-made advice to women living with HIV who want to breastfeed.

## Methods

A cross-sectional survey was setup to investigate the breastfeeding desires and decision-making of women living with HIV in the Netherlands. This survey was conducted between January 2021 and November 2021. All women living with HIV in the Netherlands could participate; there were no exclusion criteria. Some questions refer to breastfeeding desire in the past; therefore, we also included women who were >45 years of age, and were not considered of childbearing potential.

The questionnaire was administered to the women living with HIV in the Netherlands in two ways. Participants were approached by telephone or in person by HIV nurses and researchers of three Dutch University Hospitals, one general Dutch Hospital, and invited to participate. Furthermore, two Dutch patient societies distributed a link to an online version of the same questionnaire. Participants gave oral or online consent before participation, the data were stored anonymously, and appropriate local ethics committee approval was obtained.

The questionnaire was developed by the research group using input from the patient societies. The questionnaire was pilot-tested in three women living with HIV. Based on these learned preferences, women were first approached by their treating HIV nurse or physician, and the option “I do not want to share” or “I don't know” was added to nearly all questions (except some demographics). The questionnaire was developed to collect categorical data on demographics, whether participants had breastfed before, and whether the participant's had a breastfeeding desire in the past or has for the future (yes, no, or I don't know). Participants were subsequently asked to what extent different factors influence or influenced their breastfeeding decision-making on a 5-point Likert scale (completely disagree, disagree, neutral, agree, completely agree, and I don't know).

The included factors were based on previous research.^16,17^ The questionnaire ended with three specific statements investigating whether participants would be willing to comply with additional hospital visits (once a month), infant blood sampling (once a month), and willing to accept a low risk of HIV transmission (<1/100) to be able to breastfeed, based on European and Dutch treatment guidelines; these data were also collected on a 5-point Likert scale. A final question allowed women to enter free text: “If your choice to breastfeed or not was influenced by a reason not stated above, can you please share it with us?” The English questionnaire is included as Supplementary Material.

A descriptive analysis was performed with R version 3.6.2. (R Foundation for Statistical Computing, Vienna, Austria). Categorical data about breastfeeding desires were presented as a proportion. In these calculations, participants who did not answer the question or indicated it was not applicable were excluded, whereas participants who expressed “I don't know” were included. Subsequently, we summarized breastfeeding decision-making and willingness to adhere to additional monitoring in Likert bar plots using R.

To compare the different factors influencing breastfeeding decision-making, we added up the proportion of the participants who completely agreed or agree, and completely disagreed or disagreed with a specific factor and reported the two factors with the highest agreement and disagreement scores. Participants indicating “I don't know” for a specific factor were excluded from this analysis. We performed two subanalyses on the factors in Likert bar plots to check for age effect (under and above 45 years) and ethnicity (sub-Saharan origin versus Dutch origin), as these could be potential confounders.

## Results

The respondents had a mean age of 41 years (range 24–66 years), with the majority (*n* = 55, 68%; 1 unknown) under 45 years of age. Around half of the women (*n* = 43, 52%) were born in sub-Saharan Africa, approximately one-third (*n* = 26, 32%) were born in the Netherlands, whereas the minority (*n* = 13, 16%) were born in various other countries. Almost a quarter of women were pregnant at the moment of completing the survey (*n* = 19, 23%), and the majority of the participants had at least one child (*n* = 71, 87%). The minority indicated they would like to have (additional) children (*n* = 32, 39%), whereas 14 women (17%) indicated they did not know that yet.

Of the participants who indicated that it was applicable, 16/70 (23%) responded that they had breastfed their children before. Of 14 of these 16 women it was unknown whether the women were already living with HIV during breastfeeding in the past at that time, and whether breastfeeding was done in agreement with the caretaking physicians. Two women indicated they had breastfed their children while living with HIV, and in agreement with the caretaking physicians. None of the participants reported to have breastfed while living in the Netherlands without the awareness of the caretaking physician.

Next, we investigated breastfeeding desires in the past and future. Of the participants who answered the question, 34/45 (76%) expressed that they had the desire to breastfeed in the past. A similar proportion of the participants expressed the desire to breastfeed in the future: 41/58 (71%) of the women who answered the question.

**FIG. 1.**
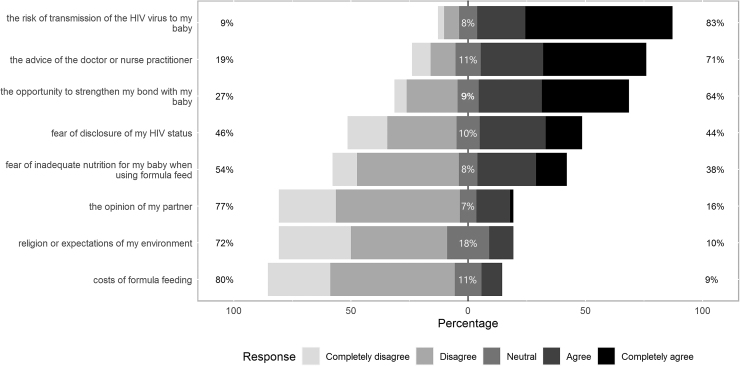
Response to the question to what extent the shown factors are/were influencing their decision to breastfeed or not. The **left** percentage depicts the percentage that completely disagrees or disagrees, whereas the percentage on the **right** represents the percentage that completely agrees or agrees. Participants could answer the questions by filling in a 5-point Likert scale and also had the option to enter “I don't know or not applicable” (not shown in the figure).

The most important factors influencing decision-making to breastfeed were the chance of transmission of HIV to the infant, the doctor's or nurse practitioner's advice and strengthening the bond with the baby. Respectively, 65/78 (83%), 53/75 (71%), and 50/78 (64%) of the participants completely agreed or agreed with these factors (Fig. 1). Costs of formula feeding the opinion of the partner and religion or expectations from environment were of least importance as, respectively, 63/79 (80%), 54/70 (77%), and 56/78 (72%) completely disagreed or disagreed with these factors. Fear of disclosure of HIV status and fear of inadequate nutrition for the baby when using formula feed were answered equally (∼50% agreed and disagreed). We did not identify an age or ethnicity effect on the factors influencing decision-making to breastfeed.

**FIG. 2.**
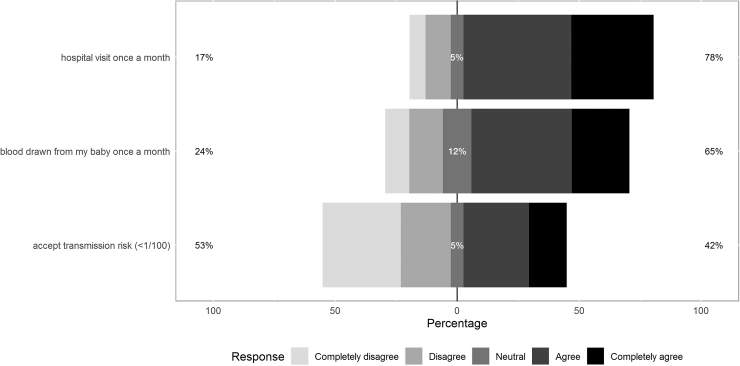
Response to the question to what extent the participants are would be willing/would have been willing to comply with the shown additional guidelines or accept the shown circumstances. The **left** percentage depicts the percentage that completely disagrees or disagrees, whereas the percentage on the **right** represents the percentage that completely agrees or agrees. Participants could answer the questions by filling in a 5-point Likert scale and also had the option to enter “I don't know or not applicable” (not shown in the figure).

Finally, we asked the participants under which circumstances or additional guidelines they would be willing or would have been willing to breastfeed. Of the participants who answered, 60/77 (78%) would have been willing/would be willing to come to the hospital once a month for a checkup if that means that she would be able breastfeed, and 33/51 (65%) of the participants would have been willing/would be willing to have blood drawn from their baby once a month (Fig. 2). Forty-one of the 78 (53%) women who answered expressed no interest in breastfeeding against a background of a transmission risk of <1/100, whereas 33/78 (42%) did. The other 5% of the participants were neutral.

Thirteen patients entered free text to answer whether other factors influenced or would influence their decision to breastfeed or not. Four patients stressed that they do not want to put the baby at risk for infection with HIV, two patients were too old to breastfeed, two patients mentioned lack of adequate information to take a decision, one patient mentioned another disease preventing breastfeeding. Other individual statements were as follows: “in my culture the only thing that makes you a mother is breastfeeding”; choice also depends on where you live and how strong stigma is; feeling an ordinary mother, HIV does not determine what is possible and what not in relation to the children; I think it's really important and for me and for my child.

## Discussion

The survey outcomes signal that a substantial part of the women living with HIV in the Netherlands have a desire to breastfeed their children. The possible MTCT risk influenced breastfeeding decision-making in most participants. Upon the specific question if they are or have been willing to breastfeed against a background of a transmission risk of <1/100, 42% of the participants expressed their interest in breastfeeding. This is comparable with the proportion of the participants that would like to breastfeed despite living with HIV in the previous studies from the United Kingdom (38%) and Switzerland (49%).^18,19^

The most important factors in decision-making around breastfeeding for women living with HIV in the Netherlands were the MTCT risk and the advice of the health care providers. These results highlight the need for more research investigating the MTCT risk in breastfeeding women living with HIV with sustained viral suppression on the current available highly effective cART regimens in high-income countries. It is currently unclear if U = U applies to transmission through breastfeeding for these women, as has been previously shown for sexual transmission and in utero transmission for women on long-term effective therapy.^21–23^ More data are needed to confirm the consensus transmission rate of <1% during breastfeeding for women who are adherent to treatment and are virologically suppressed. That the advice of the health care provider is considered important confirms and underlines the importance of health care provider and patient education and shared decision-making.^18,19^

Recent guidelines acknowledge the desire to breastfeed and mention adequate therapy and additional monitoring is required in this case.^1–3^ Our results indicate that the majority of women are willing to adhere additional hospital visits and to have monthly blood drawn from their newborns. This is in line with previous results from the United Kingdom study.^18^

The most important limitation of this survey is the relatively small sample size compared with 400 documented pregnancies in women living with HIV in the Netherlands for the past 5 years.^24^ Also, our methodology may have caused selection bias, but the distribution of the demographic origin of the participants is representative for all women with documented pregnancies from the Netherlands.^24^ Although our study results clearly indicate a significant part of the women living with HIV in the Netherlands have the desire to breastfeed, we have not collected the views of all women living with HIV in the Netherlands. Our survey mainly consisted of predefined questions and statements; however, other reasons for decision-making around breastfeeding could be included in the free text option at the end. Furthermore, the Likert scale only gives five options, which may not represent the true feeling or opinion.

## Conclusions

This study highlights the importance of having an open dialogue about breastfeeding with women living HIV. Although the possibility of HIV transmission by breastfeeding cannot be ruled out, a significant part of the women living with HIV in the Netherlands expresses the desire to breastfeed. The majority of women are willing to comply with additional monitoring to be able to breastfeed. Patient education and a tailor-made advice by the health care provider about breastfeeding or formula feeding and monitoring will enable these women to breastfeed in the safest possible way.

## Supplementary Material

Supplemental data
