# Supplementary material for: Exploring the Breastfeeding Desires and Decision-Making of Women Living with HIV in the Netherlands: Implications for Perinatal HIV Management in Developed Countries
Source: Breastfeed Med. 2023 May 17;18(5):356–61. doi: 10.1089/bfm.2023.0004 (PMC10254968; doi:10.1089/bfm.2023.0004)
Supplement: Supplemental data [file Suppl_Data.pdf]

Patient characteristics

1. How old are you?

… years

2. Where were you born? (see appendix 1 for definitions)

☐ The Netherlands
☐ Other European country (except the Netherlands)
☐ Sub-Saharan Afrika
☐ South America
☐ Caribbean
☐ South and Southeast Asia
☐ Other, namely (country):………………
☐ I don’t know / do not want to share

3. What is your marital status?

☐ Married or registered partnership
☐ Living together with partner
☐ Single
☐ Other, namely: …………………..
☐ I don’t know / do not want to share

Pregnancy

4. Are you pregnant at the moment?

☐ Yes

☐ No

☐ I don’t know / do not want to share

5. Would you like to have (another) child?

☐ Yes

☐ No

☐ I don’t know / do not want to share

6. Do you have children?

☐ Yes

☐ No, continue at question 11

☐ I don’t know / do not want to share, continue at question 11

Your choice to breastfeed

7. Since you found out you have HIV, do you have breastfed (one of) your children?

☐ Yes

☐ No, continue at question 10

☐ I don’t know / do not want to share, continue at question 10

8. If you breastfed while you knew you were living with HIV, where took that place?

☐ In the Netherlands

☐ Outside of the Netherlands, continue at question 10

☐ Both in and outside of the Netherlands

☐ I don’t know / do not want to share, continue at question 10

☐ Not applicable, continue at question 10

9. Was your Dutch HIV-doctor aware of that you were breastfeeding?

- ☐ Yes
- ☐ No
- ☐ I don't know / do not want to share

10. If you did not breastfed in the past, did you have the wish to breastfeed?

- ☐ Yes
- ☐ No
- ☐ I do not know / do not want to share
- ☐ Not applicable

11. If you would like to have (another) child, do you want to breastfeed?

- ☐ Yes
- ☐ No
- ☐ I do not know / do not want to share
- ☐ Not applicable

12. Several statements are now being presented to you. Please indicate to what extent you agree or disagree with the statement. Please express your opinion on a scale from 1 to 5. 1 means completely disagree, 3 neutral and 5 completely agree (NAP is notapplicable). There a no right or wrong answers.

12.1. For me it is important to breastfeed my baby

Completely disagree	Disagree	Neutral	Agree	Completely agree	I don't know/NAP
☐	☐	☐	☐	☐	☐

12.2. My choice to breastfeed my baby or not was / is influenced by: fear of disclosure of HIV status

Completely disagree	Disagree	Neutral	Agree	Completely agree	I don't know /NAP
☐	☐	☐	☐	☐	☐

12.3. My choice to breastfeed my baby or not was / is influenced by: the opportunity to strengthen my bond with my baby

Completely disagree	Disagree	Neutral	Agree	Completely agree	I don't know /NAP
☐	☐	☐	☐	☐	☐

12.4. My choice to breastfeed my baby or not was / is influenced by: expectations of my environment (friends/family) or my religion

Completely disagree	Disagree	Neutral	Agree	Completely agree	I don't know/NAP
☐	☐	☐	☐	☐	☐

12.5. My choice to breastfeed my baby or not was / is influenced by: fear of inadequate nutrition for my baby when using formula feed (flesmelk)

Completely disagree	Disagree	Neutral	Agree	Completely agree	I don't know/NAP
☐	☐	☐	☐	☐	☐

12.6. My choice to breastfeed my baby or not was / is influenced by: the price of formula feeding

Completely disagree	Disagree	Neutral	Agree	Completely agree	I don't know/NAP
☐	☐	☐	☐	☐	☐

12.7. My choice to breastfeed my baby or not was / is influenced by: the advice of the doctor or nurse practitioner

Completely disagree	Disagree	Neutral	Agree	Completely agree	I don't know/NAP
☐	☐	☐	☐	☐	☐

12.8. My choice to breastfeed my baby or not was / is influenced by: the risk of transmission of the HIV virus to my baby

Completely disagree	Disagree	Neutral	Agree	Completely agree	I don't know/NAP
☐	☐	☐	☐	☐	☐

12.9. My choice to breastfeed my baby or not was / is influenced by: the opinion of my partner

Completely disagree	Disagree	Neutral	Agree	Completely agree	I don't know /Nap
☐	☐	☐	☐	☐	☐

12.10 I would have been willing / would be willing to come to the hospital once a month for a check-up if that means that I would be able breastfeed

Completely disagree	Disagree	Neutral	Agree	Completely agree	I don't know/NAP
☐	☐	☐	☐	☐	☐

12.11 I would have been willing / would be willing to have blood drawn from my baby once a month if that means that I would be able breastfeed

Completely disagree	Disagree	Neutral	Agree	Completely agree	I don't know/NAP
☐	☐	☐	☐	☐	☐

12.12 I would have been willing / would be willing to accept the risk (less than 1 in 100) of transmission of the HIV virus if that means that I would be able to breastfeed

Completely disagree	Disagree	Neutral	Agree	Completely agree	I don't know/NAP
☐	☐	☐	☐	☐	☐

13. If your choice to breastfeed or not was influenced by a reason not stated above, can you please share it with us below?

End of questionnaire, thank you!

Appendix I: Countries per region

Other European country (except the Netherlands)

Albania, Andorra, Azerbaijan, Austria, Belarus, Belgium, Bosnia and Herzegovina, Bulgaria, Croatia, Czech Republic, Denmark, Germany, Estonia, Finland, France, Georgia, Greece, Hungary, Iceland, Ireland, Italy, Kazakhstan, Latvia, Liechtenstein, Lithuania, Luxembourg, Malta, Moldova, Monaco, Montenegro, North Macedonia, Norway, Poland, Portugal, Romania, Russia, San Marion, Serbia, Slovenia, Slovakia, Spain, Sweden, Switzerland, Turkey, Ukraine, United Kingdom, Vatican City

Sub-Saharan Africa:

Angola, Benin, Botswana, Burkina Faso, Burundi, Cameroon, Central African Republic, Chad, Congo-Brazzaville, Democratic Republic of Congo, Djibouti, Equatorial Guinea, Eritrea, Ethiopia, Gabon, Gambia, Ghana, Guinea, Guinea-Bissau, Ivory Coast, Kenya, Lesotho, Liberia, Madagascar, Malawi, Mali, Mauritania, Mozambique, Namibia, Niger, Nigeria, Rwanda, Senegal, Sierra Leona, Sudan, Somalia, Swaziland, Tanzania, Togo, South Africa, Southern Sudan, Uganda, Zambia, Zimbabwe

South America

Argentina, Bolivia, Brazil, Chile, Colombia, Ecuador, Guyana, Paraguay, Peru, Suriname, Uruguay, Venezuela

Caribbean

Anguilla, Antigua and Barbuda, Aruba, Bahamas, Belize, Bonaire, British Virgin Islands, Cayman Islands, Colombia, Costa Rica, Cuba, Curaçao, Dominica, Dominican Republic, French Guyana, Grenada, Guadeloupe, Guyana, Guatemala, Haiti, Honduras, Jamaica, Martinique, Mexico, Montserrat, Navassa, Nicaragua, Panama, Puerto Rico, Saba, Saint-Barthélemy, Saint Kitts and Nevis, Saint Lucia, Saint Vincent and the Grenadines, Saint Eustatius, Saint Martin, Suriname, Trinidad and Tobago , Turks and Caicos Islands, US Virgin Islands

South and Southeast Asia

Afghanistan, Bangladesh, Bhutan, Cambodia, Philippines, India, Indonesia, Laos, Maldives, Malaysia, Myanmar, Nepal, Pakistan, Sri Lanka, Thailand, Vietnam
